# Nonpathological Extracellular Amyloid Is Present during Normal Epididymal Sperm Maturation

**DOI:** 10.1371/journal.pone.0036394

**Published:** 2012-05-03

**Authors:** Sandra Whelly, Seethal Johnson, Jonathan Powell, Clinton Borchardt, Mary Catherine Hastert, Gail A. Cornwall

**Affiliations:** 1 Department of Cell Biology and Biochemistry, Texas Tech University Health Sciences Center, Lubbock, Texas, United States of America; 2 Department of Biology, Imaging Center, Texas Tech University, Lubbock, Texas, United States of America; University of Maryland, United States of America

## Abstract

Amyloids are aggregated proteins characterized by a specific cross-β-sheet structure and are typically associated with neurodegenerative diseases including Alzheimer's disease. Recently, however, several nonpathological amyloids have been found in intracellular organelles of normal mammalian tissues suggesting that amyloid may also carry out biological functions. We previously have shown that the epididymal cystatin CRES (cystatin-related epididymal spermatogenic), *cst8*, a reproductive-specific member of the cystatin superfamily of cysteine protease inhibitors, forms amyloid in vitro suggesting that CRES amyloid may also form in vivo within the epididymal lumen. Here we show that amyloid structures containing CRES are a component of the normal mouse epididymal lumen without any apparent cytotoxic effects on spermatozoa and that these structures change along the length of the tubule. These studies suggest the presence of a functional amyloid structure that may carry out roles in sperm maturation or maintenance of the luminal milieu and which itself may undergo maturational changes along the epididymis. In contrast to previous examples of functional amyloid which were intracellular, our studies now show that nonpathological/functional amyloid can also be extracellular. The presence of an extracellular and nonpathological amyloid in the epididymis suggests that similar amyloid structures may be present in other organ systems and may carry out distinctive tissue-specific functions.

## Introduction

Amyloids are aggregated proteins with a specific cross-β-sheet structure that typically are associated with neurodegenerative diseases such as Alzheimer's and Parkinson's disease. Several nonpathological amyloids have recently been found in intracellular organelles of normal mammalian tissues suggesting that amyloid may also carry out biological functions. These include in melanosomes the Pmel protein which forms an amyloid structure involved in the synthesis of melanin [1] and the storage of several hormones as amyloids in the pituitary gland [2]. The formation of amyloid is a multi-step process in which proteins progress from soluble oligomers to fibrils with the oligomeric amyloid thought to be the cytotoxic species [3]. Because functional and pathological amyloids are structurally similar, in normal tissues amyloidogenesis must be controlled to avoid cytotoxicity.

The epididymis is a highly convoluted tubule through which spermatozoa must pass after exiting the testis in order to acquire the functional capacities of progressive motility and the ability to fertilize an oocyte. This maturation process requires the interaction of spermatozoa with proteins synthesized and secreted into the lumen in a region-dependent manner by the epididymal epithelium [4]. In addition to maturation, the epididymal luminal environment plays a critical role in the protection of the spermatozoa since the viability of these cells must be maintained in order for perpetuation of the species to occur. Because greater than 90% of the water is absorbed from the fluid by the epithelium as spermatozoa enter into the epididymis, the epididymal luminal environment surrounding the spermatozoa is thought to be a dense medium rich in particulate material of unknown function.

We have previously demonstrated that the reproductive cystatin CRES (cystatin-related epididymal spermatogenic) (*cst8*), a member of the cystatin superfamily of cysteine protease inhibitors, is synthesized and secreted by the most proximal part of the mouse epididymis (proximal caput, region 1) and thus may be involved in the sperm maturation process [5]. We have also shown that the monomeric 14 and N-glycosylated 19 kDa forms of CRES are present within the lumen of the proximal caput but in more distal epididymal regions disappear with a concomitant appearance of CRES in high molecular mass structures [6]. Immunofluorescence analyses also showed that in the distal epididymis CRES localized to punctuate-type structures of unknown function [7]. In vitro studies of the recombinant CRES protein demonstrated that it has the capacity to readily form oligomeric and fibrillar amyloid-like structures characteristic of those present in pathological conditions such as Alzheimer's disease [6]. In this regard CRES is similar to the L68Q mutant form of human cystatin C which readily forms amyloid and is the causative factor of hereditary cystatin C amyloid angiopathy (HCCAA) in the Icelandic population [8]. Our observation that CRES forms amyloid in vitro raised the question of whether CRES might also form amyloid in vivo and thus explain the transition of monomer to high molecular mass structures. Therefore, the present studies were carried out to determine if amyloid-like structures are present in the normal mouse epididymal lumen and, in particular, whether CRES contributes to the formation of these structures. Herein we show that CRES amyloid is a component of the normal epididymal luminal environment and thus the first example of a mammalian extracellular nonpathological amyloid.

## Materials and Methods

### Animals/Ethics Statement

CD1 retired breeder male mice were purchased from Charles River Laboratories (Wilmington, MA). *Cst8* 129SvEv/B6 gene knockout and wildtype mice were bred in house. Mice were maintained under a constant 12 h light/12 h dark cycle with food and water *ad libitum*. All animal studies were conducted in accordance with the NIH Guidelines for the Care and Use of Experimental Animals using a protocol approved by the TTUHSC IACUC.

### Isolation of epididymal luminal fluid and pellet fractions

Epididymides were sectioned into five regions with 1, proximal caput; 2, midcaput; 3, distal caput; 4, corpus; and 5, cauda or into caput (regions 1–3) and corpus-cauda (regions 4–5). The tissue sections were minced in PBS and luminal contents allowed to disperse for 15 minutes. The suspension containing spermatozoa and luminal proteins was centrifuged at 500×g for 5 min to pellet spermatozoa (pellet 1) and any epithelial cells and the supernatant removed and centrifuged again at 500×g to remove any remaining cellular material. The supernatant representing the total luminal fluid protein (soluble and particulate) was either used directly in experiments or underwent differential centrifugation to separate out particulate material of varying molecular mass. This included centrifugation of the total luminal fluid first at 5000×g for 10 min (pellet 2), followed by centrifugation of the supernatant at 15,000×g for 10 min (pellet 3) followed by centrifugation of the supernatant in a Beckman ultracentrifuge at 250,000×g for 1 hour (pellet 4). All pellets were resuspended in PBS and stored on ice. In some experiments, pellet 4 was washed in PBS and extracted with 90% DMSO at room temperature for 1.5 hours prior to centrifugation at 250,000×g for 1 hour. In other experiments, after centrifugation to remove cellular material (pellet 1), the supernatant was centrifuged at 250,000×g for 1 hour combining pellets 2–4 into one high speed pellet. The final supernatant from the last centrifugation was designated the supernatant fraction.

### Dot blot and filter trap assays

For dot blot analysis, 10 µg of protein from the supernatant and high speed pellet fractions from each of the five epididymal regions was spotted on to nitrocellulose (Biotrace, Pall Corp, Ann Arbor, MI) and incubated with either the A11 or OC antibodies (Millipore, Billeria, MA) at 1∶3000 or 1∶5000, respectively in TBST overnight at 4°C as described previously [6].

For filter trap assays, cellulose acetate membrane (Whatman OE66, 0.2 µm) prewet in water was placed on top of PVDF membrane (activated in methanol and then equilibrated in water) which was placed on top of prewet filter paper in a dot blot apparatus. PBS was added to each well and vacuum applied. Samples (15 µg) were then loaded followed by a PBS wash. The membranes were immediately placed in 3% milk/TBST (0.2% Tween) to block for 1 hr at room temperature followed by incubation with an affinity purified rabbit anti-mouse CRES antibody 1 µg/ml at 4°C overnight. The specificity of the CRES antibody for the CRES antigen has been previously established [5]. The blots were washed 2× in TBST, incubated with goat anti-rabbit HRP-labeled secondary antibody (Thermo Scientific, Rockford, IL) for 2 hrs at room temperature, washed in TBST and incubated with chemiluminescence reagent (BioRad, Hercules, CA).

### Thioflavin S and Congo Red staining

Pellet samples were spread on to Superfrost Plus microscope slides and allowed to air dry. Slides were incubated in 0.1% Thioflavin S, prepared in water and filtered prior to use, for 2 hours at room temperature in the dark. The slides were washed 4× in water, 2× in 50% ethanol, followed by 2× in water and mounted with VectaMount AQ (Vector Laboratories, Burlingame, CA). For Congo Red, air dried slides were stained following the method of Puchtler [9] overnight in a humidified chamber. Slides were rinsed 5× with water, 3×95% ethanol, 4×100% ethanol, 1× xylene and mounted with VectaMount (Vector Laboratories, Burlingame, CA). For plate assays, samples were incubated with 20 µM thioflavin T and fluorescence determined with a Synergy HT plate reader (BioTek, Winooski, VT) with excitation at 450 nm and emission at 485 nm.

### Negative stain electron microscopy

Samples (5 µl) were either spotted on to formvar/carbon coated 200 mesh nickel grids (Ted Pella, Redding, CA) or the grid was floated on the sample for 1 min. The sample was wicked off with filter paper, washed for 1 min with water, stained with 2% uranyl acetate for 1 min, and washed again with water for 1 min. Samples were examined with a Hitachi 8100 electron microscope operating at an excitation voltage of 75 kV.

### CRES/Thioflavin S colocalization

Slides were stained with 0.05% Thioflavin S for 2 hours following the protocol described above. After the last water wash, slides were transferred to a humid plastic container and incubated with 3.7% formaldehyde in PBS for 30 minutes at room temperature. The fixative was wicked off and replaced with 1% Triton X-100 for 3 minutes after which the slides were washed 3× in PBS containing 0.1% Tween (PBST) and then incubated with an affinity purified rabbit anti-mouse CRES antibody 1 µg/ml in PBST/10% goat serum at room temperature for 1 hour followed by incubation at 4C° overnight. The slides were washed with PBST 5× and incubated with a goat anti-rabbit Alexa Fluor 594 conjugated secondary antibody (Invitrogen, Grand Island, NY) (1∶250) in PBST/10% goat serum for 2 hours at room temperature in the dark. Slides were washed 5× in PBST, 1× water and mounted with VectaMount AQ. Slides were examined using a Zeiss microscope equipped with epifluorescence with excitation at 425/40 nm and emission at 475 nm for Thioflavin S and 560/40 nm excitation and 610 nm emission for Alexafluor 594.

### Western blot analysis

Western blot analysis was carried out as described previously using 15% Tris-glycine SDS-PAGE gels (Criterion, Bio-Rad, Hercules, CA) [6].

### Transmission electron microscopy

A high speed pellet from the caput epididymis was resuspended in a small volume of Histogel and processed for Epon embedding following standard protocols.

### Protein aggregation disease (PAD) ligand capture

Luminal fluid was centrifuged to remove spermatozoa and the resulting supernatant was incubated with PAD ligand for 2 hours at room temperature and washed following the manufacturer's protocol (Seprion, Microsens Biotechnologies, CalBioreagents, San Mateo, CA), and eluted with 1× Lammeli (2% SDS, 4% β-mercaptoethanol) at 95°C for 5 minutes.

### X-ray diffraction

Pellet 4 was isolated from the epididymis in 10 mM HEPES, pH 7.4 with final resuspension in water. The solution was pulled up into a 0.7 mm quartz capillary tube and allowed to air dry. Samples were exposed to X-rays at 1.54 Å for 15–30 minutes at room temperature using a Rigaku Screen machine (Rigaku, The Woodlands, TX).

## Results

### Amyloid in the epididymis

**Figure 1:**
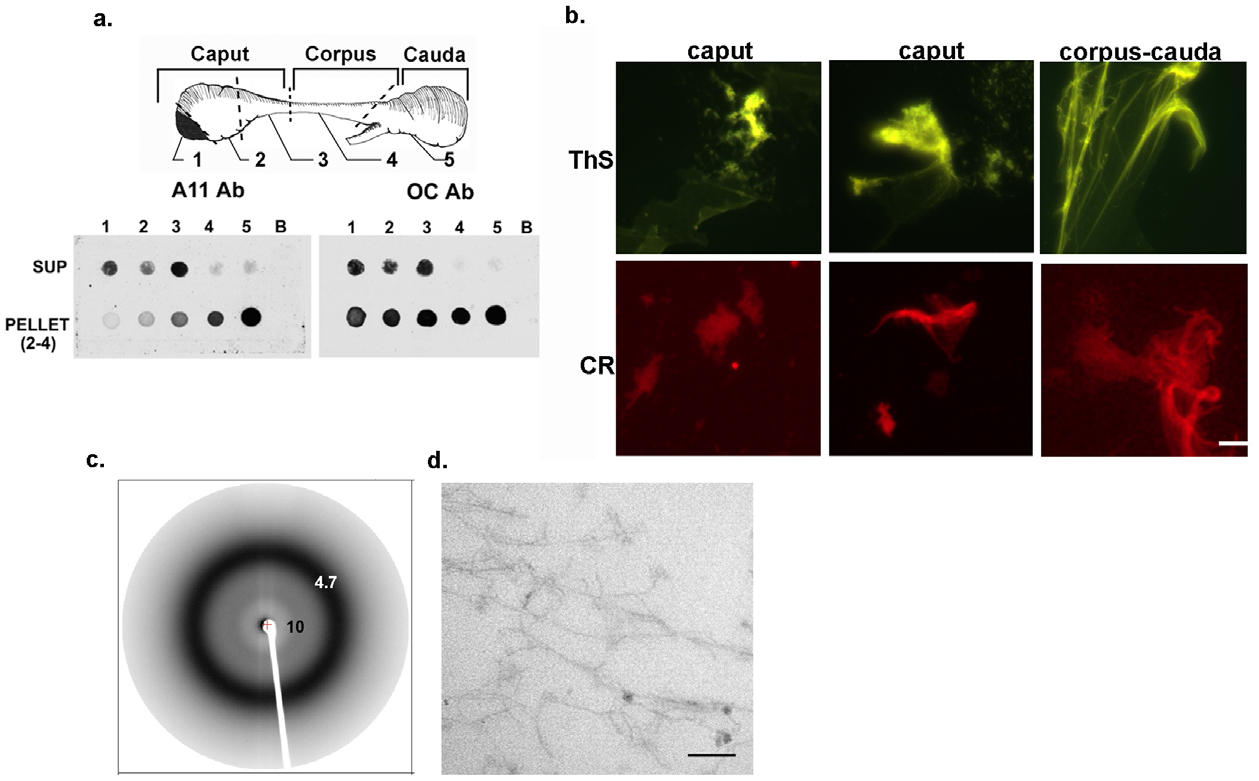
Amyloid structures in the mouse epididymal lumen. a) Dot blot analysis using All and OC antibodies to detect the oligomeric and fibrillar amyloid structures, respectively, in supernatant and high speed pellet fractions isolated from the five regions of the mouse epididymis. b) Thioflavin S (ThS) and Congo Red (CR) bound to similar structures including a film-like material (third panel) in the high speed pellet isolated from the caput (regions 1–3) and corpus-cauda (regions 4–5) luminal fluid from the mouse epididymis. Bar, 5 µm. c) X-ray diffraction of a 250,000×g pellet (pellet 4) isolated from the corpus-cauda luminal fluid. d) Transmission electron microscopy of an Epon embedded high speed pellet isolated from the caput epididymal luminal fluid. Bar, 100 µm.

To determine whether amyloid structures are present in the epididymal lumen a variety of approaches were used. First, conformation-dependent antibodies, A11 and OC, which recognize oligomeric and fibrillar forms of amyloid, respectively [10] were used in dot blot analysis to characterize the forms of amyloid in the epididymal fluid. Following removal of spermatozoa, a supernatant and a high speed pellet fraction isolated from the luminal fluid from each of the five regions of the mouse epididymis were examined. These studies revealed the presence of amyloid-like structures in both the supernatant and pellet fractions. Region-dependent changes in the composition of the structures were also observed with the caput (regions 1–3) containing oligomeric (A11) and fibrillar (OC) forms of amyloid in both the supernatant and pellet while the corpus-cauda (regions 4–5) contained amyloid only in the pellet (Figure 1a). This suggests that a transition or maturation of the amyloid structures may occur along the length of the tubule with the cauda epididymis containing primarily insoluble forms of amyloid.

**Figure 2:**
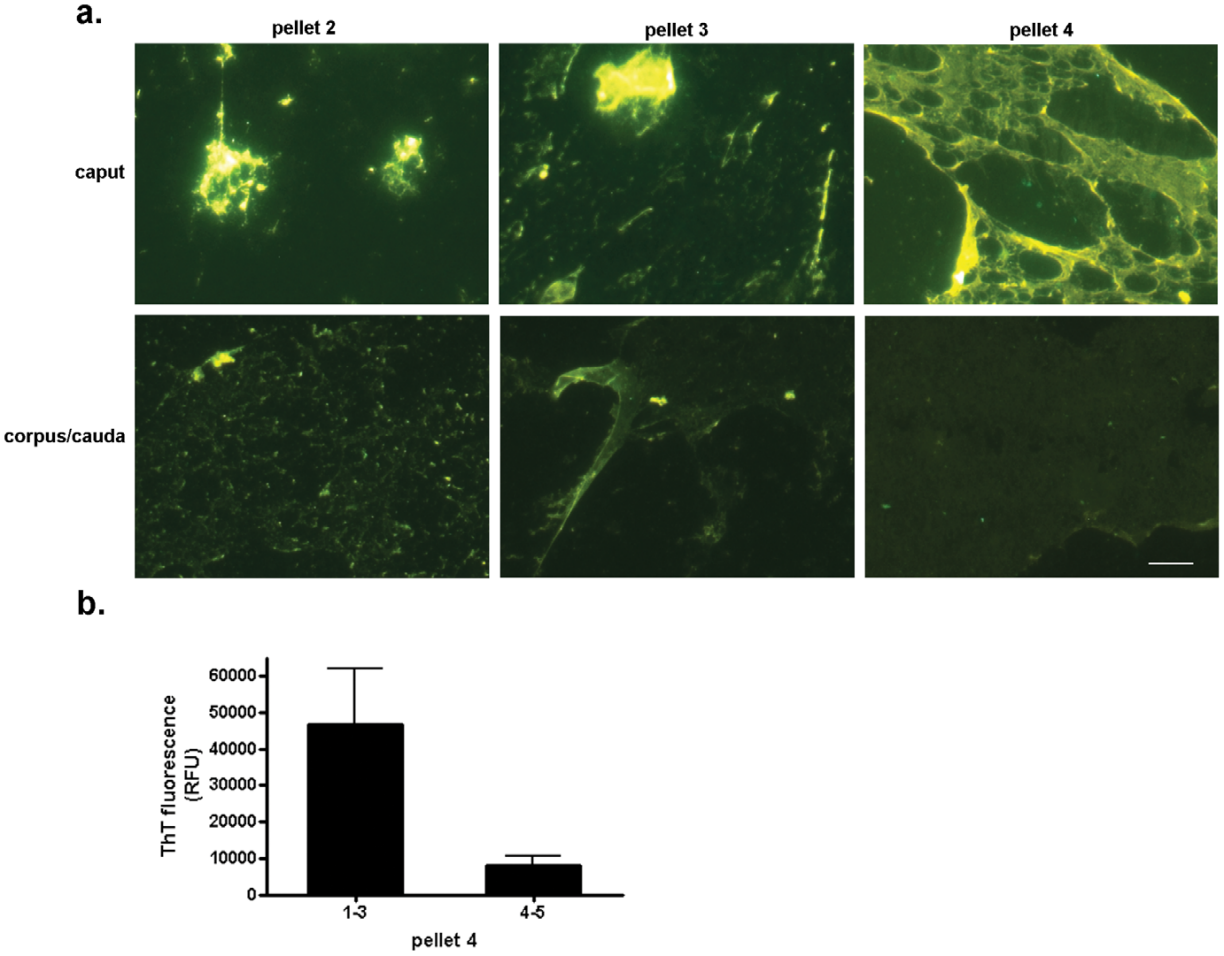
Regional changes in thioflavin staining in the epididymis. a) Sequential centrifugation was carried out to isolate particulate material of varying molecular mass from the caput and corpus-cauda luminal fluid after first removal of spermatozoa by centrifugation at 500×g (pellet 1). Pellet 2, 5000×g; pellet 3, 15000×g; pellet 4, 250,000×g. Samples were air dried on a slide and stained with 0.1% thioflavin S. All images were captured with the same exposure times. Bar, 5 µm. b) 15 µg of protein from the caput (regions 1–3) and corpus-cauda (regions 4–5) pellet 4 was examined for thioflavin T fluorescence in a plate assay. Mean±SEM of three experiments.

To confirm the presence of amyloid, the conformation-dependent dyes thioflavin S and Congo Red, which bind primarily to fibrillar forms, were used to stain the structures in the high speed pellet fractions which were then examined by fluorescence microscopy. Both dyes bound to similar structures from the caput and corpus-cauda epididymis, including a unique film-like material, suggesting these structures were composed of cross- β-sheets typical of amyloid (Figure 1b). Further fractionation by sequential centrifugation and characterization of the pellet fractions showed that in the caput epididymis a variety of large aggregate structures that were thioflavin S positive were associated with low centrifugation speeds (pellet 2, 5000×g; pellet 3, 15000×g) while the film-like material was associated with a 250,000×g pellet (pellet 4) (Figure 2a). In the cauda epididymis, a mesh of small aggregate material was detected in pellet 2 (5000×g) while a beginning film-like material was detected in pellet 3 (15000×g) and a dense film-like material was predominant in pellet 4 (250,000×g). Although the cauda material was also thioflavin S positive, supporting its possessing cross β-sheet structure, the relative thioflavin S levels were profoundly reduced compared to that in the caput epididymis (Figure 2a). This suggests that during epididymal transit the amyloid structures are changing perhaps acquiring a different conformation in the cauda epididymis that prevents thioflavin S staining. In support, the thioflavin T fluorescence of pellet 4, as determined by plate assay, showed an approximately 6-fold reduction in fluorescence in the cauda epididymis compared to that in the caput (Figure 2b). Because pellet 4 contained an enriched population of the film-like amyloid rather than a heterogeneous mix of structures as observed in the other pellets, we focused on characterizing this amyloid structure in subsequent experiments.

To examine the crystallographic properties of the structures in pellet 4, X-ray diffraction was performed on samples isolated from the corpus-cauda epididymis. A major reflection at 4.7 Å, representing spacing between strands in a β-sheet, and a more diffuse reflection at approximately10 Å, representing spacing between β-sheets, were observed (Figure 1c). Both reflections are characteristic of cross-β-sheet structure present in amyloid [11]. Finally, the high speed pellet from the caput epididymis was examined by standard transmission electron microscopy after embedding in Epon to preserve structural integrity. Fibrils approximately 10 nm in diameter typical of amyloid fibrils were detected (Figure 1d).

**Figure 3:**
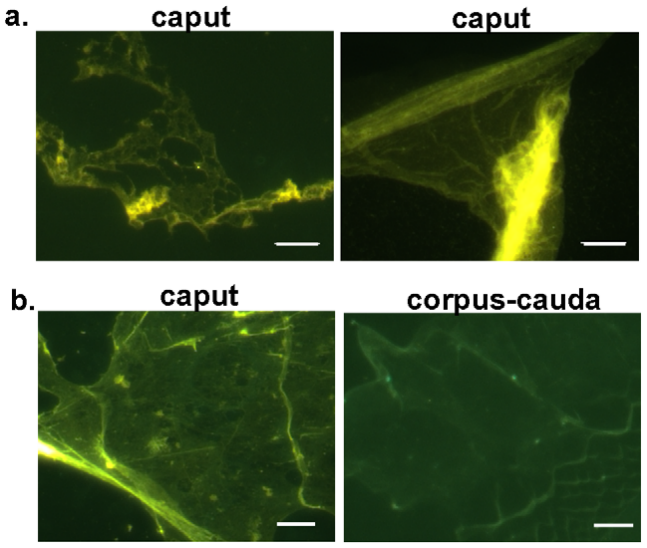
Thioflavin S staining of epididymal luminal fluid. a) Caput luminal fluid was centrifuged to pellet spermatozoa and the resulting supernatant dried on a slide and stained with thioflavin S. Representative structures are shown. b) Luminal fluid was isolated after puncturing the caput and corpus-cauda epididymal tubules with a needle and allowing contents to disperse. After centrifugation to remove spermatozoa, the supernatant was dried on a slide and stained with thioflavin S. Bar, 5 µm.

Additional experiments were next carried out to determine whether the thioflavin S positive film-like material was a normal component of the luminal fluid and not created as a result of the isolation procedure. The thioflavin S positive film-like material was present in whole luminal fluid that underwent only a single low speed centrifugation spin (500×g) to remove spermatozoa demonstrating that this structure was not formed as a result of ultracentrifugation (Figure 3a). To rule out that the film-like material was due to contamination that occurred during the mincing of the tissue, both caput and corpus-cauda epididymal tubules were punctured to allow dispersion of the contents which were then examined by thioflavin S staining. The thioflavin S positive film-like material was again observed suggesting that it is a normal component of the luminal fluid (Figure 3b).

**Figure 4:**
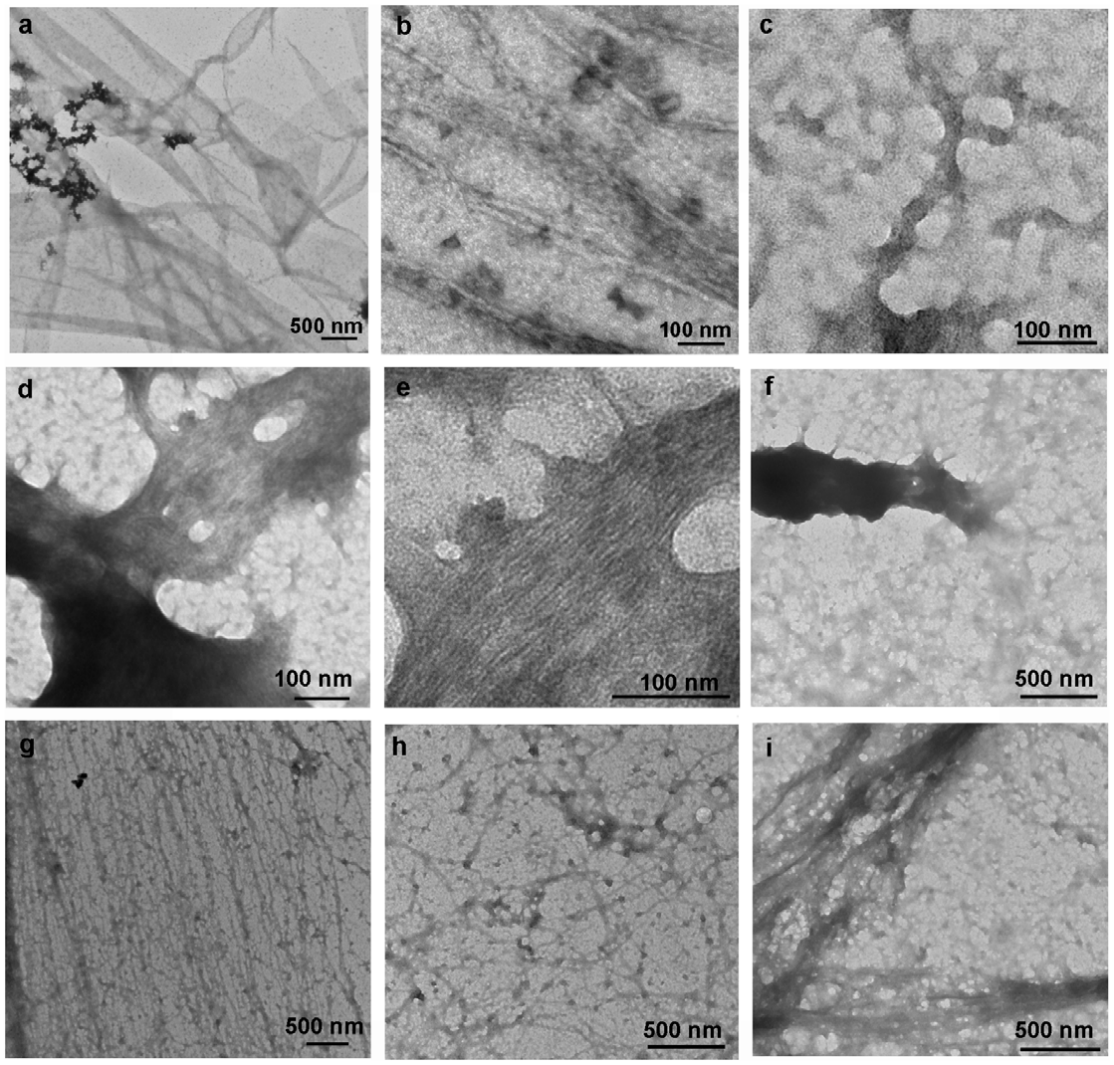
Negative stain electron microscopic analysis of amyloid structures from the caput and corpus-cauda luminal fluid. The 250,000×g pellet 4 prepared from the caput (a–e) and corpus-cauda epididymal luminal fluid (f–i) was spotted on a grid, stained with 2% uranyl acetate, and examined using a Hitatchi 8100 electron microscope. Panel e shows a higher magnification of panel d.

Negative stain electron microscopy was next carried out to characterize the amyloid structures in pellet 4 from the caput and corpus-cauda epididymidis. While similar structures were detected in both epididymal regions, the caput contained more ribbon-like and branched polygon structures (Figure 4a,c) in addition to fibrillar structures (Figure 4b) that were present within the ribbons, while the corpus-cauda contained arrays of fibrils and filaments (Figure 4g–i). Several of these structures appeared to have amorphous material associated with them possibly representative of protein-protein interactions between amyloid and nonamyloid forming proteins (Figure 4c). Large prion-like structures that were composed of stacked fibrils (Figure 4d, e) or a meshwork (Figure 4f) were also observed in both regions. Taken together, these studies show that amyloid structures are present within the normal mouse epididymal lumen. Furthermore, there are region-dependent changes along the epididymis in the composition of the amyloid structures which may reflect either different amyloids or an amyloid that is undergoing a transition to a different structure.

### CRES amyloid in the epididymis

**Figure 5:**
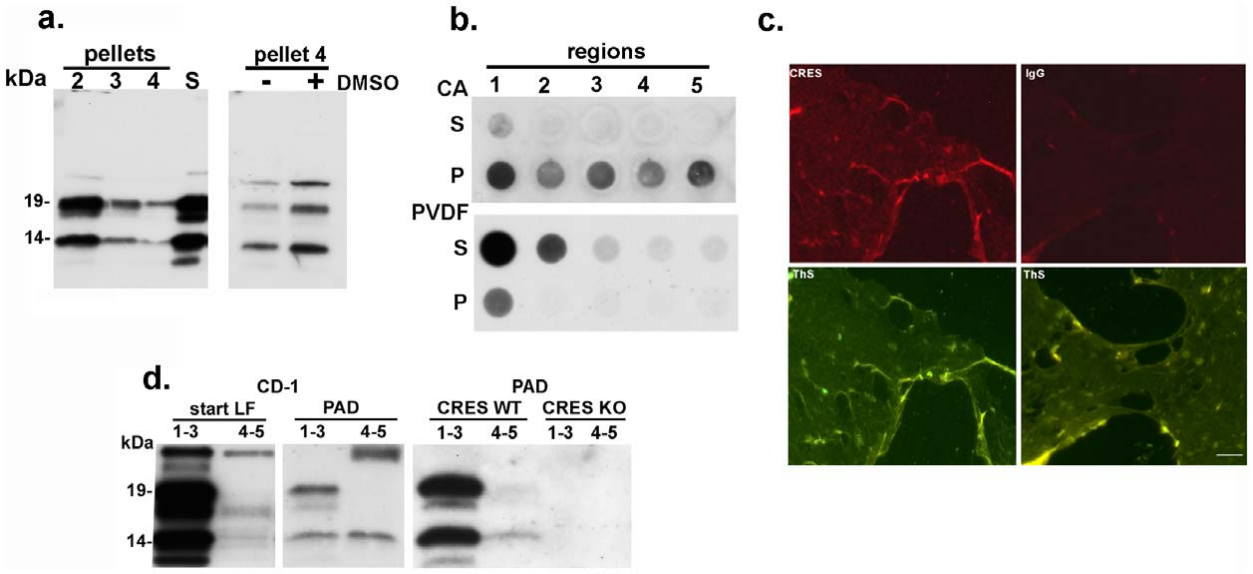
CRES amyloid in the mouse epididymal lumen. a) Left, Western blot analysis of CRES in pellet and supernatant (S) fractions obtained after sequential centrifugation of the luminal fluid from caput region 1. Right, pretreatment of pellet 4 from caput region 1 with DMSO (+) prior to Western blot analysis of CRES. b) Filter trap assay of the supernatant (S) and high speed pellet (P) isolated from the luminal fluid from each of the five epididymal regions. Samples were spotted onto cellulose acetate (CA) overlying PVDF membrane followed by incubation with affinity purified CRES antibody. c) Pellet 4 samples from the caput luminal fluid were dried on a slide and stained with 0.05% thioflavin S followed by incubation with an affinity purified CRES antibody or IgG (control) followed by an Alexafluor 594 secondary antibody. d) Left panel, incubation of luminal fluid (LF) containing particulate material from the caput (regions 1–3)and corpus-cauda (regions 4–5) from CD-1 mice with protein aggregation disease (PAD) ligand followed by Western blot analysis using an affinity purified CRES antibody. Right panel, incubation of PAD ligand with luminal fluid isolated from the caput or corpus-cauda from CRES WT (wildtype) and KO (knockout) mice followed by Western blot analysis using affinity purified CRES antibody.

Studies were first carried out to examine the distribution of CRES within the supernatant and pellet fractions isolated from the luminal fluid. Although a proportion of CRES remained in the supernatant (S) from caput region 1, CRES was also detected in pellets 2–4 implying its presence in particulate structures (Figure 5a). DMSO has been used in studies of amyloid forming proteins as a means to disrupt aggregated β-sheets [12]. Pretreatment of pellet 4 with DMSO prior to Western blot analysis resulted in the appearance of additional monomeric forms of CRES suggesting that a proportion of the protein in this fraction is in a higher order structure that is resistant to SDS but which is reversed by DMSO (Figure 5a). Filter trap assays supported the Western blot experiments since in the high speed pellet fractions from all five epididymal regions CRES was detected on the cellulose acetate membrane suggestive of its presence in a high molecular mass structure that is trapped and unable to pass through the membrane while CRES in the supernatant readily passed through the filter to bind to the underlying PVDF membrane (Figure 5b). The population of CRES in the supernatant likely represents CRES monomers as well as soluble oligomers, precursors to the later stage amyloid protofibrils and fibrils [3].

To determine whether CRES amyloid was present in the epididymis, colocalization studies were carried out. Immunofluorescence analysis using an affinity-purified CRES antibody showed that CRES colocalized with the thioflavin S positive film-like material in pellet 4 from the caput epididymis suggesting CRES is in an amyloid structure (Figure 5c). However, we were unable to detect CRES in the film-like material in pellet 4 isolated from the cauda epididymis (data not shown). We next incubated luminal fluid samples with a polymeric ligand (protein aggregation disease (PAD)) reagent consisting of repeating charged and hydrophobic groups that interact with similar repeating groups on aggregated proteins, and which has previously been shown to selectively capture prion proteins [13] and oligomeric and fibrillar amyloid from a Huntington's disease mouse model [14]. As shown in Figure 5d, the PAD ligand captured a proportion of CRES from both the caput and corpus-cauda luminal fluid suggesting its presence in the epididymal lumen as an amyloid structure. Similar results were obtained when PAD ligand was used to capture CRES from the luminal fluid from a CRES WT but not from a CRES KO mouse model (Figure 5d). The 129 SvEv mouse strain expresses higher levels of CRES than the CD-1 mice used in all the other experiments and thus more CRES protein was captured from the CRES WT by the PAD ligand in these studies.

## Discussion

### Amyloid in the epididymis

Taken together, our studies suggest that within the epididymis CRES amyloid is a part of the normal extracellular environment with no obvious detriment to the maturing spermatozoa and thus may be an example of an amyloid that carries out biological roles within the epididymal lumen. Specifically, using techniques that are common to the amyloid field, including the use of conformation-dependent antibodies and dyes, X-ray diffraction, and electron microscopy we showed the presence of amyloid in the epididymal lumen. Furthermore, colocalization studies using an affinity purified CRES antibody and thioflavin S staining and PAD ligand pulldown assays showed that CRES contributes to the formation of amyloid within the epididymal lumen. The epididymal amyloid may provide a critical infrastructure for the transport of luminal proteins to the sperm surface as part of the maturation process, serve as an organizational center for protein interactions within the lumen, and/or as a means of extracellular quality control.

### Maturational changes in amyloid

Changes in amyloid were also detected along the epididymal tubule which could reflect a maturational progression of the amyloid into different structures or distinct region-specific populations of amyloid. Indeed, region-dependent changes in proteins are a hallmark of the epididymis and contribute to the formation of region-dependent luminal microenvironments that are essential for sperm maturation [15], [16]. The caput epididymis is the most metabolically active region and secretes the bulk of the proteins found in the lumen. Many of these proteins remain in the lumen and are found in the cauda epididymis while other proteins are selectively removed or modified during epididymal transit. Therefore, the apparent maturational change in the amyloid structures from the caput to the cauda is consistent with epididymal function and may be integral to normal maturation events.

Specifically, while both the caput and cauda epididymis contained a film-like amyloid material in the 250,000×g pellet 4, the intensity of the thioflavin S and thioflavin T fluorescence was much lower in pellet 4, as well as in all pellets, from the cauda epididymis compared to that in the caput suggesting that there was less amyloid in the cauda compared to the caput epididymis. However, studies of amyloidogenic proteins have shown that a lack of thioflavin fluorescence can also be associated with an amyloid that has acquired a higher ordered structure that is not favorable for thioflavin binding [17]–[19]. To address this, studies are currently ongoing to determine if the film-like material in pellet 4 as well as material in other pellets from the cauda epididymis can be reversed to structures that show increased thioflavin binding.

In parallel with the maturational change in amyloid along the epididymis, our ability to detect CRES amyloid also changed from the caput to the cauda epididymis. Strong CRES immunofluorescence colocalized with strong thioflavin S fluorescence in the caput pellet fractions while our inability to detect CRES in the cauda pellets coincided with decreased thioflavin S fluorescence. However, filter trap assays clearly suggest that CRES is present in the cauda pellet fractions and PAD pulldown experiments showed the presence of CRES amyloid in the cauda lumen. Thus it may be that the mechanisms that result in reduced thioflavin S binding contribute to our decreased ability to detect CRES in the cauda epididymis. An alternate possibility to the formation of higher ordered structures contributing to reduced thioflavin S binding may be that in the cauda epididymis other modifications such as transglutaminase crosslinking may have altered the amyloid structures making them less able to bind thioflavin [6], that the proteins contributing to the amyloid structures in the cauda may be distinct from those in the caput and thus thioflavin binding properties are different, or that amyloid structures are actually being reversed within the cauda lumen, perhaps as a prelude to removal from the lumen.

### Control of amyloidogenesis in the epididymis

Previous examples of nonpathological/functional amyloid have been found sequestered within cellular organelles thus providing the cell some protection from cytotoxicity that can arise during amyloidogenesis [20]. Indeed, although it was previously thought that amyloid fibrils caused cell death, more recent work suggests that it is the oligomeric forms of amyloid, precursors to fibrils, that are cytotoxic by virtue of their ability to form pores/channels in cell membranes and disrupt cell homeostasis [3], [21]. The control of Pmel amyloid formation is thought to occur at multiple levels in addition to sequestration within the melanosome, including the requirement for proteolytic processing by proprotein convertases [22] and rapid kinetics of amyloid fibril formation avoiding cytotoxic oligomeric intermediates [1]. Similarly, pituitary hormones form amyloid in the secretory granules and this process is thought to be controlled by proteolytic processing as well as possible interactions with helper proteins [2]. Our studies showing the presence of amyloid in the epididymal extracellular environment suggests that unique mechanisms must be in place to control and/or protect against cytotoxicity during amyloidogenesis. This is especially important within the epididymis since amyloid is present in the same cellular compartment as spermatozoa and damage to these cells must be avoided. It is possible that interactions with chaperones may help to control cytotoxicity. Cystatin C is also present within the mouse epididymal lumen and has been shown to form amyloid in vitro [23]. Another possibility is that interactions between several cystatin proteins form the amyloid structures present within the lumen and this coordinated and regulated interaction may be a mechanism to control amyloid formation and avoid cytotoxicity. This would be similar to that which occurs in bacteria where the interaction of several secreted curli protein family members mediates the formation of a functional amyloid structure [24].

Our studies show that nonpathological/functional amyloids can be extracellular as well as intracellular which is similar to amyloids associated with disease. These studies add to the growing list of nonpathological/functional amyloids that have been detected within mammals supporting the idea that these structures may indeed be carrying out diverse biological functions in many organ systems including the reproductive tract. Further studies of epididymal amyloid including mechanisms that control its formation will provide valuable insight towards understanding uncontrolled amyloid formation as occurs in many neurodegenerative diseases.
